# Validity of the telematic Fugl Meyer assessment scale – upper extremity (TFMA-UE) Spanish version

**DOI:** 10.3389/fneur.2023.1226192

**Published:** 2023-08-10

**Authors:** Rocío Llamas-Ramos, Inés Llamas-Ramos, Fátima Pérez-Robledo, Juan Luis Sánchez-González, Beatriz María Bermejo-Gil, Elisa Frutos-Bernal, Ana María Martín-Nogueras

**Affiliations:** ^1^Department of Nursery and Physiotherapy, Faculty of Nursery and Physiotherapy, Universidad de Salamanca, Salamanca, Spain; ^2^University Hospital of Salamanca, Salamanca, Spain; ^3^Department of Statistics, Facultad de Medicina, Universidad de Salamanca, Campus Miguel de Unamuno, Salamanca, Spain

**Keywords:** Fugl Meyer assessment scale, physiotherapy, telematic, assessment, acquire brain injury

## Abstract

**Background:**

Telematic assistance has become indispensable in recent years. The increased prevalence of Acquired brain injury and the sequels it causes, requires long–lasting multidisciplinary treatments. Validated tools to assess the evolution of the disabilities and limitations of this pathology are essential to individualize and prescribe adapted treatments. The aim has been to create the telematic version of the Fugl Meyer Assessment-Upper Extremity Motor Function (TFMA-UE) Spanish scale and its adaptation to the remote assessment of neurologic patients.

**Methods:**

An adapted scale was designed based on the Fugl Meyer Assessment scale-telematic version (FMA-TV): TFMA-UE. This scale is composed by 21 items which evaluate the upper extremity motor function. Physiotherapists trained in this tool, evaluate the results obtained from applying the two versions (on-site and telematic) to compare the results.

**Results:**

TFMA-UE was administered to 30 patients with acquired brain injury. It was applied on site and through the web platform selected by the patients in two different days. Patients completed all the scale in an easily way without help. The exploratory and confirmatory factor analysis confirmed a factorial structure with a factor (76.08% of the variance). The Cronbach’s internal consistency index obtained was 0.98 and the weight kappa index used to measure agreement between the two versions was 0.78 which represents substantial agreement.

**Conclusion:**

The Telematic Fugl Meyer Assessment-Upper Extremity Motor Function (TFMA-UE) scale is a viable, useful and easy to apply tool that allows the upper extremity motor function assessment of Acquired Brain Injury patients.

## 1. Introduction

Telerehabilitation requires validated assessment tools to determine the patient’s functionality in order to establish objectives and choose treatment tools.

Acquired brain injury (ABI) is the third leading cause of death and the most common cause of disability and dependency even among young adults (1). The main ABI etiologies are traumatic brain injury (TBI) and stroke, although it includes other causes unrelated to congenital or degenerative disorders (2). The incidence of ABI due to stroke is twice that those caused by TBI. In Spain, the population of people with ABI is increasing and currently there are an estimated 500,000 people with ABI, of which 78% are caused by stroke (3, 4), and between 50,000 and 75,000 people have suffered a TBI (5), it is also being the first cause of hospitalization due to neurological pathology (6). The disability it generates is very high, as well as the healthcare expenses it entails. In 2020, The United States, Sweden, and Spain were the 3 countries with the highest average expenditure (US dollars: 59,900, 52,725, and 41,950 respectively) (7).

The consequences or sequelae present in ABI are very heterogeneous, which requires multidisciplinary teams to apply individualized treatments (8, 9). The most prevalent are paralysis (unilateral), language and speech impairment, orientation and coordination limitations and sensory disability, resulting in functional limitation, decreased independence and a need for constant care (10). These motor, sensory, cognitive and language deficits affect patients’ daily activities, impacting their quality of life and participation in the community (11, 12). They may also suffer from anxiety, fatigue and/or depression (13), sometimes being unable to return to their jobs (14).

The management of these patients has improved considerably in recent years reducing mortality and morbidity thanks to the approach in the acute phase and the prevention of risk factors. The increased survival of these patients and the presence of sequelae require long-term rehabilitation interventions (11, 15). Neurorehabilitation techniques have proven to be effective in improving balance and functionality, obtaining results in as little as 7 days (16). Since 2019, society and therapies have had to adapt, largely as a consequence of the pandemic generated by COVID-19 that forced the incorporation of the use of communication technologies in healthcare practice, both for prevalent and incident cases (17).

Telematic treatments are proving useful in reducing the time from the onset of symptoms to the start of on-site treatments, with satisfactory audiovisual quality and high inter-rater reliability, which, in short, reduces the need for hospital transfers (18). This system is particularly relevant when, despite an increase in the incidence of stroke, after COVID-19 there has been less stroke admission patients to the emergency department, which could be due to fear of contagion on the part of patients and/or family members (19). Therefore, in their study, Dafer et al. (19) established guidelines with recommendations for the management of these stroke patients from prehospital consultations to more advanced phases such as rehabilitation treatment. These authors advocate the use of telemedicine after a previous in-hospital assessment, since the functional prognosis of the patients will depend on the rehabilitation treatment (19).

This current and innovative care system requires the adaptation and validation of assessment, diagnostic and treatment tools. The availability of standardized assessment scales facilitates the recording and documentation of symptoms, makes it possible to specify specific and feasible treatment objectives and to select the most appropriate tools. For neurological patients one of the most widely used scales is the Fugl Meyer scale (11, 20) and its original version has already been adapted to Spanish (21). It has a short version (22), and others for the upper and lower extremity (23). Its telematic feasibility has recently been demonstrated with satisfactory results (24).

The aim of the present study was the validation of the Fugl Meyer Assessment - Upper Extremity Motor Function - telematic version (TFMA-UE) scale in patients with ABI, taking into account the clinical needs detected and the high prevalence of sequelae in the upper limb in these patients.

## 2. Methods

### 2.1. Design

Cross-sectional study for the adaptation and validation of the Fugl Meyer Assessment - Upper Extremity Motor Function scale - telematic version (TFMA - UE).

### 2.2. Sample

The sample was composed by neurological patients from the “Asociación de Daño Cerebral Adquirido” from Salamanca (ASDACE) who voluntarily decided to participate after being informed of the objectives and procedures of the study. Recruitment of the sample was carried out between December 2021 and April 2022. All patients with ABI were included if they had the facilities for telematic connection and it was feasible to use them. Patients with cognitive alterations, difficulties in following orders or interacting during a video call were excluded.

### 2.3. Instruments

The Fugl Meyer – Upper Extremity Motor Function scale - telematic version (TFMA - UE) has been established from the telematic version Fugl Meyer assessment scale (FMT-TV) (24) which in turn was concretized from the Fugl Meyer scale validated to Spanish (21).

The original Fugl Meyer scale (25) was created to assess the functional status of patients who had suffered a stroke composed by 5 domains: upper extremity motor function, lower extremity motor function, balance, sensitivity, range and joint pain and contains 113 items and 226 points. This scale has been validated into Spanish (21), adapted to telematic version, and its viability has been demonstrated (24). The FMA -TV scale presents a total score of 92 points and differs from the original in those items that require the presence of the therapist and its remote assessment is compromised. Thus, the FMA -TV scale presents 4 domains: upper extremity motor function, lower extremity motor function, balance and pain. It does not include items related to the assessment of reflexes, resisted movements, or movements requiring objects or assistance.

The TFMA-UE scale is constructed from the upper extremity motor function domain of the FMA-TV. This scale in comparison to the original Fugl Meyer (25) does not contain: the 3 items related to reflexes (biceps, triceps and finger flexors); the 2 items of resisted wrist movements; 5 items of the grasp section related to grasp (hook grasp., thumb adduction, pincer grasp/opposition, cylinder grasp and spherical grasp) and 2 coordination items (tremor and dysmetria). The TFMA-UE scale consists of 21 items and 42 points: 15 items related to the upper extremity/shoulder, 3 wrist items, 2 hand items and 1 coordination item. Each item is valued in the same way as in the original scale: from 0 to 2, being 0 the impossibility of performing the movement, 1 the beginning of the movement or incomplete performance but not performed correctly and 2 the correct performance of the movement (Annex 1).

### 2.4. Procedure

All subjects were evaluated on-site using the Fugl Meyer scale - Spanish version (24) (upper extremity motor function dimension) by different physiotherapists trained in the use of the tool, in the facilities of the Faculty of Nursing and Physiotherapy of the University of Salamanca. Between 2 and 5 days later, the telematic evaluation was carried out using the TFMA-UE scale for which the platform chosen by each participant according to their preferences was used: Google Meet, Facetime, Whatsapp or Microsoft Teams.

The patients remained seated in a chair, with their backs against the backrest and the soles of their feet flat on the floor. The camera was placed at a distance that allowed a full view of both upper extremities. A caregiver was always present to assist in the placement of the camera and to reassure the patient in case of any unforeseen event.

Five physiotherapists undertook joint training to ensure the comprehension of all the items and a similar evaluation in all cases. The same physiotherapists were in charge of performing the on-site and the telematic evaluations.

### 2.5. Statistical analysis

The psychometric properties of the TFMA-UE were evaluated. Cronbach alpha coefficient was used to assess reliability and exploratory factor analysis (EFA) and confirmatory factor analysis (CFA) were performed to assess the validity. SPSS Statistics version 28.0 and SPSS Amos version 26.0 were employed.

Sample adequacy indices were obtained, indicating a good fit of the data. Therefore, to discover the underlying structure of the items EFA was employed, and given the ordinal nature of the data, the method employed was the unweight least squares based on the polychoric correlation matrix (26). The screen test was evaluated, so that the components located above the curve of the sedimentation graph are taken into account. Those items whose factor loads were greater than 0.40 and which did not significantly load more than one factor were selected.

The CFA model suggested by EFA was tested. The maximum likelihood estimation was chosen because of the ordinal scales of the data (27). To evaluate the goodness of fit of the model, it was verified that the correction of $S-B_{\chi^{2}}$ on the degree of freedom was less than 3; the Tucker-Lewis Index (TLI) and the Comparative Fit Index (CFI) (28) had values greater than or equal to 0.9. Furthermore, the Akaike Information Criterion (AIC) was evaluated, knowing that the lower its value, the more parsimonious the model is (29).

The weight kappa index was used to evaluate the concordance or reproducibility of the two instruments (original and telematic). The kappa results are interpreted as follows: values between 0.01 and 0.20 as none to slight, 0.21 and 0.40 as fair, 0.41 and 0.60 as moderate, 0.61 and 0.80 as substantial and between 0.81 and 1.00 as almost perfect agreement (30).

### 2.6. Ethical considerations

The study had the approval of the ethics committee of the University of Salamanca with registration number: 630, registration in Clinical.Trial.org with ID: NCT04670315 and the Declaration of Helsinki has been taken into account. All participants were previously informed of the objectives of the study and signed an informed consent.

## 3. Results

The sample consisted of 30 patients with a mean age of 67.01, CI 95% [63.36–70.66] and 4.22 CI 95% [3.29–5.16] years of evolution since the diagnosis of ABI. 66.7% were male (Table 1).

**Table 1:** Descriptive data of the sample.

		Patients *n* = 30
Age* (years)		67.01 [63.36–70.66]
Time since diagnosis* (years)		4.22 [3.29–5.16]
Civil status**	Single	0 (0.0)
	Married	26 (86.7)
	Divorced	1 (3.3)
	Widowed	3 (10.0)
Sex**	Male	20 (66.7)
	Female	10 (33.3)
Dominant side**	Right	28 (93.3)
	Left	2 (6.7)
Affected side**	Right	14 (46.7)
	Left	16 (53.3)
Occupation**	Primary sector	4 (13.3)
	Secondary sector	4 (13.3)
	Service sector	19 (63.3)
	Others	3 (10.0)
Educational level**	Primary	8 (26.7)
	Secondary	5 (13.7)
	High School	6 (20.0)
	University	10 (33.3)
	No studies	1 (3.3)
Brumstrom stage**	2	3 (10.0)
	3	4 (13.3)
	4	4 (13.3)
	5	6 (20.0)
	6	10 (33.3)
	No evaluable	3 (10.0)

*Mean [95% confidence interval] **number (percentage).

Table 2 shows the mean scores obtained by the patients for each of the sections of the telematic version together with the mean total score.

**Table 2:** Scores obtained by sections and total score of telematic version.

Section	Item	Mean	SD
Flexor synergy	Shoulder retraction	1.03	0.77
	Shoulder elevation	1.40	0.62
	Shoulder abduction	1.33	0.71
	Shoulder external rotation	1	0.74
	Elbow flexion	1.63	0.72
	Forearm supination	1.33	0.88
Extensor synergy	Shoulder adduction/internal rotation	1.67	0.61
	Elbow extension	1.57	0.77
	Forearm pronation	1.60	0.68
Volitional movement mixing synergies	Hand to lumbar spine	1.53	0.82
	Shoulder flexion 0°–90°	1.30	0.79
	Pronation-supination	1.30	0.84
Volitional movement with little or no synergy	Shoulder abduction 0–90°	1.27	0.83
	Shoulder flexion 90°–180°	0.93	0.79
	Pronation/supination	1	0.83
Wrist	Repeated dorsiflexion/volar flexion (elbow 90°)	1.17	0.87
	Repeated dorsiflexion/volar flexion (elbow 0°)	0.97	0.81
	Circumduction	1.10	0.85
Hand	Mass flexion	1.60	0.72
	Mass extension	1.33	0.88
Coordination	Time	1.27	0.87
Total score		27.33	13.91

The sample presented a good fit, with Bartlett’s sphericity test being significant (*p* < 0.001) and the Kaiser–Meyer–Olkin test (KMO) of 0.884. The exploratory factor analysis suggested a three-factor solution. The model was redefined by performing a second EFA after eliminating the items that did not meet the retention criteria: items 1 (shoulder retraction), 2 (shoulder elevation) and 3 (shoulder abduction). This resulted in an unifactorial solution that explained 76.08% of the variance, in accordance with the factorial structure of the original scale (Table 3).

**Table 3:** Exploratory factor analysis: loadings, means standard deviations of the TFMA – UE.

ITEM	Loading	M	SD
Shoulder external rotation	0.80	1	0.74
Elbow flexion	0.88	1.63	0.72
Forearm supination	0.87	1.33	0.88
Shoulder adduction/internal rotation	0.81	1.67	0.61
Elbow extension	0.93	1.57	0.77
Forearm pronation	0.86	1.60	0.68
Hand to lumbar spine	0.91	1.53	0.82
Shoulder flexion 0°- 90°	0.90	1.30	0.79
Pronation-supination	0.91	1.30	0.84
Shoulder abduction 0 - 90°	0.92	1.27	0.83
Shoulder flexion 90° - 180°	0.78	0.93	0.79
Pronation/supination	0.86	1	0.83
Repeated dorsiflexion / volar flexion (elbow 90°)	0.85	1.17	0.87
Repeated dorsiflexion / volar flexion (elbow 0°)	0.81	0.97	0.81
Circumduction	0.87	1.10	0.85
Mass flexion	0.91	1.60	0.72
Mass extension	0.90	1.33	0.88
Time	0.92	1.27	0.87

**Figure 1:**
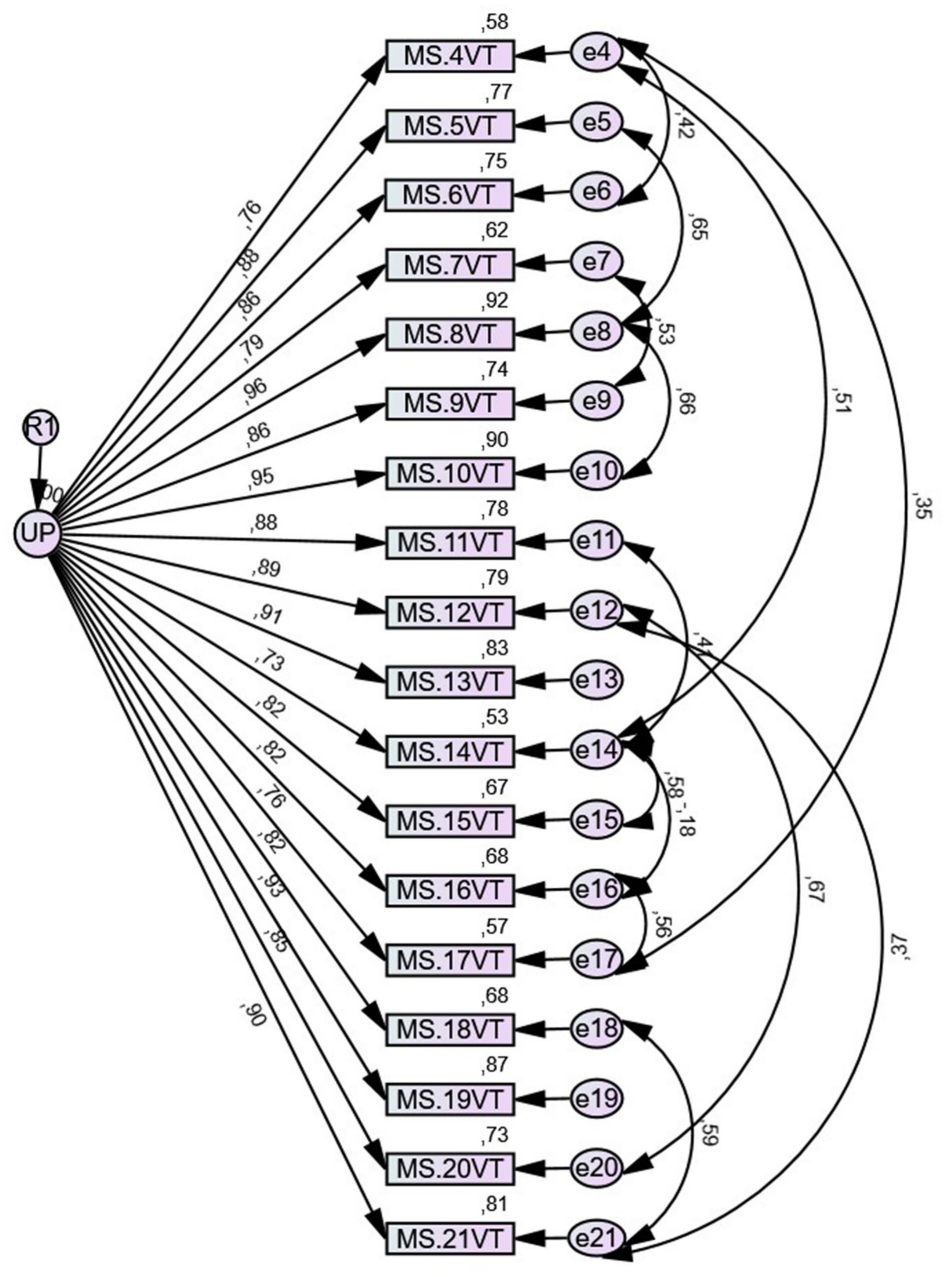
Path diagram with standardized weights and measurement errors for each item of the scale.

A CFA was performed using the maximum likelihood method. The one-factor model obtained through EFA and composed of 18 items has a good fit (Table 4). The factor loadings range from 0.71 to 0.91 (Figure 1). The reliability of the scale was found to be high, with Cronbach’s Alpha Coefficient equal to 0.98.

**Table 4:** Confirmatory factor analysis of the TFMA – UE.

$S-B_{\chi^{2}}/gl$	Goodness-of-fit test	TLI	CFI	AIC
1.70	$\chi^{2}=207.093$ gl = 122, *p* = 0.00	0.878	0.902	305.093

TLI, Tucker-Lewis Index; GFI, Goodness-of-Fit Index; CFI, Comparative Fit Index; RMSEA, root mean square error of approximation; AIC, Akaike information criterion.

The analysis of concordance was established using the weighted kappa coefficient whose values range from 0.54 to 0.924 with an average value of 0.78 which could be considered substantial agreement. The two items (shoulder elevation and shoulder abduction) with a coefficient lower than 0.60 were the ones excluded in the exploratory and confirmatory factor analysis (Table 5).

**Table 5:** Weight Kappa statistic values.

Section	Item	Weight Kappa	CI [95%]
Flexor synergy	Shoulder external rotation	0.75	[0.57; 0.94]
	Elbow flexion	0.88	[0.72; 1,04]
	Forearm supination	0.72	[0.52; 0.91]
Extensor synergy	Shoulder adduction/internal rotation	0.80	[0.57; 1.03]
	Elbow extension	0.86	[0.70; 1.02]
	Forearm pronation	0.66	[0.41–0.91]
Volitional movement mixing synergies	Hand to lumbar spine	0.90	[0.77–1.03]
	Shoulder flexion 0°–90°	0.71	[0.50–0.92]
	Pronation-supination	0.92	[0.81–1.03]
Volitional movement with little or no synergy	Shoulder abduction 0–90°	0.92	[0.81–1.03]
	Shoulder flexion 90°–180°	0.75	[0.57–0.93]
	Pronation/supination	0.92	[0.82–1.08]
Wrist	Repeated dorsiflexion/volar flexion (elbow 90°)	0.71	[0.52–0.90]
	Repeated dorsiflexion/volar flexion (elbow 0°)	0.76	[0.56–0.97]
	Circumduction	0.78	[0.63–0.94]
Hand	Mass flexion	0.77	[0.59–0.95]
	Mass extension	0.74	[0.55–0.93]
Coordination	Time	0.85	[0.71–0.99]

*p* < 0.01.

**Figure 2:**
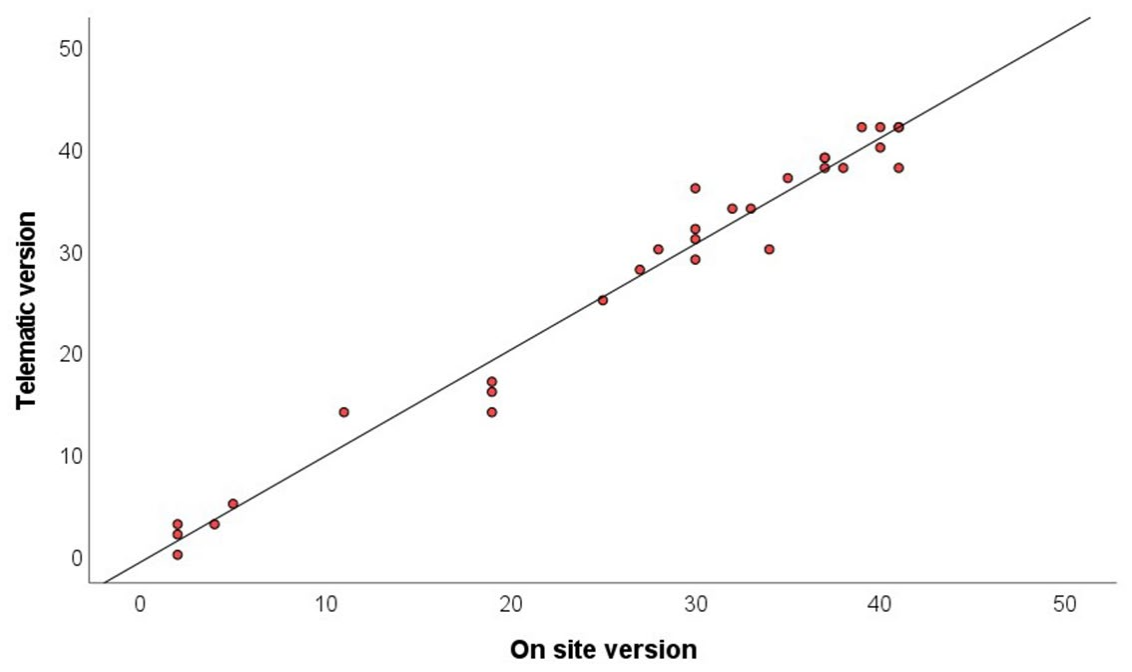
Scatterplot showing the concordance between on-site and telematic version of Fugl-Meyer assessment of upper extremity motor function total score.

Figure 2 presents a scatterplot of the total scores obtained in the on-site and telematic version of the Fugl-Meyer upper extremity motor function scale. A high correlation was obtained between the overall scores obtained with the two instruments, the Pearson correlation coefficient being *r* = 0.987 (*p* < 0.01).

## 4. Discussion

This study carried out the validation of the telematic version of the Fugl Meyer Assessment Scale - upper extremity motor function (TFMA - UE) obtaining good results in the psychometric properties of reliability and validity. It is essential to have validated tools to assess the functionality of patients with ABI telematically, to establish individualized treatment goals adapted to the stages of evolution, and to select the most appropriate treatment tools.

In 2016, there were 13.7 million strokes and more than 80 million stroke survivors worldwide (31). Rehabilitation treatments can reduce disability and dependency, improve the quality of life of people suffering ABI, their caregivers, and the national economy (32–34).

The addition of telematic care for people with ABI will reduce waiting times for access to rehabilitation treatment, as well as increase the possibility of access for people with geographical or personal difficulties who cannot travel to on-site centers. The sequelae or impairments that occur in the upper extremity in a person with ABI generate functional limitations among which are weakness or paralysis, loss of sensitivity and pain (35–39), which usually become chronic. The average rehabilitation time reflected in the literature varies according to the studies, establishing durations between 2 and 6 weeks, from 3 to 7 day-weeks and from 90 to 1,288 min-weeks without a clear consensus on which is the best therapeutic option. However, a positive effect has been found between rehabilitation time and upper extremity motor impairment (39). The impairment presented by these patients is not always unique and several impairments are often present at the same time, which makes it difficult to adapt treatments. Incorporating telematic assessment and treatment strategies will allow optimizing the prescription in these patients (38).

The Fugl Meyer scale (25), based on the sequential recovery of motor function by Twitchell and Brunnstrom (38), represents one of the most widely used scales in motor recovery after stroke (40) and its clinical utility is well supported (41). Our research team has long experience evaluating patients with ABI with the Spanish version of this scale. The interruption of treatment during the Covid-19 pandemic made it necessary to implement telematic care strategies, perceiving the need for instruments to evaluate the functionality of these patients (24). For months the team members checked which items were feasible to be applied by video call and after a long process of debate the FMA-TV scale was established and its feasibility was proven (24). The FMA-TV scale does not contain items related to reflexes, sensitivity, range and pain and neither have they been included in the validation of our TFMA - UE version. The removal of these items does not interfere with functional outcomes since according to Reener et al. (42) wrist extensors and flexors and fingers are good predictors of upper extremity motor function (42). Woodbury et al. (23) had already proposed the elimination of the 3 reflex-related items. The TFMA - UE scale contains movement speed, a direct, objective and reliable kinematic measure of movement abilities as demonstrated by several authors (43, 44). The TFMA - UE has a total score of 42 points compared to 66 points for the upper extremity motor function dimension of the FMA scale original version.

For the validation of our scale we used the original version of the Fugl Meyer - Upper extremity scale (23). After eliminating items 1, 2, 3, the EFA analysis found a single dimension, the same as the original version (25), with 76.08% of the variability explained. This solution was validated by CFA, obtaining a good fit. In addition, the reliability of the scale was checked by means of the Cronbach Alpha Coefficient and its concordance with the original scale by means of the weighted kappa coefficient, obtaining good results in both cases. The lack of concordance in the shoulder elevation and shoulder abduction items can be explained by the alteration in the anticipatory postural adjustments (APA). After suffering a stroke, there is an impairment in the cortico-spontine networks and an alteration in the information coming from the supplementary motor area, related to the temporal component of the APAs. This may be the reason why patients present a delay in the APAs (45) and alteration in normal movement patterns. In the scapula-humeral stabilizers this alteration results in worse stability and orientation of the shoulder and/or trunk during movement (46), generating excessive trunk displacement and decreased shoulder flexion and adduction along with reduced elbow extension (50). These movement alterations make it difficult to assess selective movements, as many times compensatory or substitute movements occur together (47), and this fact is more difficult to discern through a screen.

Recent studies have investigated the telematics application of this scale. On the one hand, Liz et al. (48) have implemented a study to verify possible errors in the telematic application of the Fugl Meyer scale, concluding that it presents excellent intra- and inter-rater reliability, which makes it valid for the evaluation of these patients. On the other hand, Carmona et al. (49) designed a modified version of the Upper extremity Fugl Meyer Scale with good results, unfortunately these authors did not analyze the psychometric properties of the scale like in our study. Both studies are in line of our investigation and supports our finding that the TFMA-UE could be used as an alternative tool evaluation for this population and also could be implemented in patients with arm disabilities.

Although this study has allowed the validation of the proposed scale, it is not without some limitations, the sample size being the main one. It is important to point out that this sample size is conditioned by the prevalence of ABI. For all these reasons, it would be interesting to carry out future studies that include a larger sample size.

## 5. Conclusion

The telematic version of the Fugl Meyer Assessment - upper extremity motor function (TFMA - UE) scale has demonstrated a high degree of validity and reliability being a suitable instrument for functional assessment of the upper extremity telematically in patients who have suffered an ABI.

## Data availability statement

The raw data supporting the conclusions of this article will be made available by the authors, without undue reservation.

## Ethics statement

The studies involving human participants were reviewed and approved by the ethics committee of the University of Salamanca, obtained with registration number: 630. The patients/participants provided their written informed consent to participate in this study.

## Author contributions

RL-R, IL-R, and FP-R researched literature and conceived the study. RL-R, IL-R, FP-R, JS-G, BB-G, EF-B, and AM-N were involved in protocol development, gaining ethical approval, patient recruitment and data analysis. RL-R and IL-R wrote the first draft of the manuscript. All authors contributed to the article and approved the submitted version.

## Funding

This research has been funded by the CPFCYL. Number code: INV2023-39.

## Conflict of interest

The authors declare that the research was conducted in the absence of any commercial or financial relationships that could be construed as a potential conflict of interest.

## Publisher’s note

All claims expressed in this article are solely those of the authors and do not necessarily represent those of their affiliated organizations, or those of the publisher, the editors and the reviewers. Any product that may be evaluated in this article, or claim that may be made by its manufacturer, is not guaranteed or endorsed by the publisher.
