# Supplementary material for: Validity of the telematic Fugl Meyer assessment scale – upper extremity (TFMA-UE) Spanish version
Source: Front Neurol. 2023 Aug 10;14:1226192. doi: 10.3389/fneur.2023.1226192 (PMC10449578; doi:10.3389/fneur.2023.1226192)
Supplement: Supplementary file 1 [file Table_1.DOCX]

Supplementary Material

Validity of the Telematic Fugl Meyer Assessment – UE (TFMA-UE) Spanish Version

**Rocío Llamas-Ramos*, Inés Llamas-Ramos, Fátima Pérez-Robledo, Juan Luis Sánchez-González, Beatriz María Bermejo-Gil, Elisa Frutos Bernal, Ana María Martín-Nogueras**

*** Correspondence:** Inés Llamas-Ramos: [inesllamas@usal.es](mailto:inesllamas@usal.es)

# Supplementary Data: TFMA- UE

Código ID: Fecha de valoración:

**VALORACIÓN DE LA FUNCIÓN MOTORA EN EL MIEMBRO SUPERIOR**

| **SECCIÓN** | **ÍTEM** | **PUNTUACIÓN** |
| --- | --- | --- |
| **EXTREMIDAD SUPERIOR**  (posición sentada) | | |
| **I. SINERGIA FLEXORA:**  0: no realiza la acción  1: la realiza parcialmente  2:la realiza perfectamente | **1. Elevación escapular** |  |
|  | **2. Retracción de hombro** |  |
|  | **3. Abducción de hombro** |  |
|  | **4. Rotación externa del hombro** |  |
|  | **5. Flexión del codo** |  |
|  | **6. Supinación del antebrazo** |  |
| **II. SINERGIA EXTENSORA**  0: no realiza la acción  1: la realiza parcialmente  2: la realiza perfectamente | **7. Aducción/rotación interna del hombro** |  |
|  | **8. Extensión del codo** |  |
|  | **9. Pronación del antebrazo** |  |
| **III. MOVIMIENTOS COMBINANDO**  **SINERGIAS** | **10. Mano hacia columna lumbar**  (0) – no puede realizar la acción  (1) – Realiza la acción parcialmente (la mano debe pasar espina iliaca anterosuperior)  (2)- la realiza perfectamente |  |
|  | **11. Hombro flexionado a 90º, codo a 0º**  (0) – El brazo se abduce inmediatamente, o el codo se flexiona al inicio del movimiento  (1) – Abducción o flexión del codo ocurre en una fase más tardía del movimiento  (2)- Realiza la acción perfectamente |  |
|  | **12. Pronación/supinación del antebrazo, codo en**  **90º, hombro en 0º.**  (0) – Posición correcta del hombro y no se puede conseguir la flexión del codo ni la pronación/supinación.  (1) – La pronación o supinación activa pueden realizarse parcialmente (el hombro y codo están posicionados correctamente).  (2)- Realiza la acción perfectamente |  |
| **IV. MOVIMIENTOS QUE NO COMBINAN SINERGIAS** | **13. Abducción de hombro hasta 90º, codo en 0º,**  **antebrazo pronado**  (0) – Se produce flexión inicial en el codo, o alguna tendencia a la pronación del antebrazo.  (1) – El movimiento se puede realizar parcialmente, o, si durante el movimiento, el codo se flexiona, o el antebrazo no se puede mantener en pronación.  (2)- Se realiza perfectamente. |  |
|  | **14. Flexión del hombro entre 90-180º, codo en 0º y**  **antebrazo en posición intermedia**  (0) – Se inicia flexión del codo, o se realiza abducción del hombro  (1) – La flexión de codo o la abducción de hombro ocurre durante la flexión del hombro (en las fases más tardías del movimiento).  (2) - Se realiza perfectamente |  |
|  | **15. Pronación/supinación del antebrazo, codo en**  **0º y hombro en 30-90º de flexión**  (0) – La supinación y pronación no puede realizarse.  (1) – El codo y el hombro correctamente posicionados se sitúan adecuadamente pero la supinación se realiza en un rango limitado  (2) - Se realiza perfectamente. |  |
| **MUÑECA** | **16. Flexión dorsal y palmar de muñeca (flexión/extensión, codo en 90º y hombro en 0º)**  (0) – No existen movimiento voluntarios de flexión palmar  (1) – El paciente no puede completar activamente el rango total de movimiento de la muñeca  (2) – Impecable, completando de manera repetitiva el rango total de movimiento articular. |  |
|  | **17. Flexión dorsal y palmar de muñeca flexión/extensión, codo en 0º y hombro en 30º)74**  (0) – No existen movimientos voluntarios de flexión palmar  (1) – El paciente no puede completar activamente el rango total  de movimiento de la muñeca  (2) – Impecable. |  |
|  | **18. Movimientos circulares con la muñeca**  (0) – No se puede realizar  (1) – Movimiento errático o circunducción incompleta  (2) – Movimiento impecable. |  |
| **MANO** | **19. Flexión de dedos**  (0) – No existe flexión  (1) – Alguna flexión pero no re realiza el movimiento completo  (2) – Flexión (activa) completa (comparada con la mano no  afectada) |  |
|  | **20. Extensión de dedos**  (0) – No existe extensión  (1) – El paciente puede realizar un agarre flexor activo pero no el movimiento completo  (2) – Extensión (activa) completa (comparada con la mano no afectada) |  |
| **COORDINACIÓN** | **21. Velocidad**  (0) – La actividad se realiza en más de 6 segundos más lento que con la mano no afecta  (1) – Entre 2 y 5.9 segundos más lento que con la mano no afectada  (2) – Menos de 2 segundos de diferencia |  |
| PUNTUACIÓN TOTAL | /42 puntos | |

| **PUNTUACIÓN TOTAL** | **/42 puntos** |
| --- | --- |

**
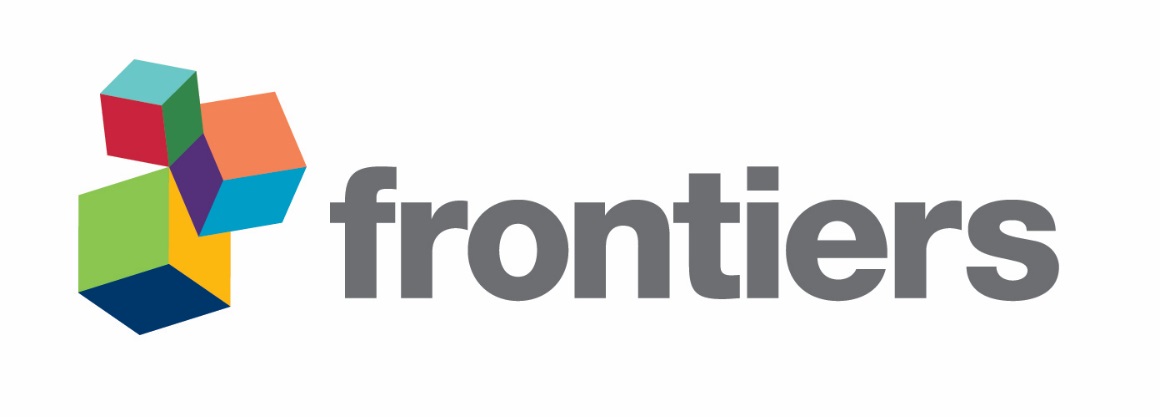
**
